# Effects of ‘SPRAT’ programme for dietary and lifestyle education to improve psychosomatic symptoms and dietary habits among adolescents: a cluster randomised controlled trial

**DOI:** 10.1186/s12889-022-12832-7

**Published:** 2022-03-08

**Authors:** Junko Watanabe, Mariko Watanabe, Kazue Yamaoka, Misa Adachi, Asuka Suzuki, Toshiro Tango, Visiting Professor

**Affiliations:** 1 The Department of Nutrition Management, Minami Kyushu University, Miyazaki, Japan; 2 Showa Women’s University, Tokyo, Japan; 3 Teikyo University Graduate School of Public Health, 2-11-1, Kaga Itabashi-ku, 1738605 Tokyo, Japan; 4 Nutrition Support Network LLC, Kanagawa, Japan; 5 Center for Medical Statistics, Tokyo, Japan

**Keywords:** Subjective psychosomatic symptoms, Adolescents, Lifestyle modification, School-based, Effectiveness, Cluster randomisation

## Abstract

**Background:**

Dietary and lifestyle modifications to reduce subjective psychosomatic symptoms (SPS) have become an important topic worldwide. We developed a school-based dietary and lifestyle education programme that involved parents/guardians in reducing SPS in adolescents (SPRAT). The programme encouraged parents/guardians to participate in adolescents’ healthy dietary and lifestyle modifications to reduce SPS, increase enjoyment of school life, and foster appropriate dietary intake. This study evaluated the effectiveness of SPRAT in reducing SPS and in altering dietary behaviour among adolescents.

**Methods:**

A 6-month cluster randomised controlled trial using SPRAT and the usual school programme (control) was performed. Participants were middle school students in Japan who provided informed consent. Outcomes were SPS scores assessed at baseline and 2, 4, and 6 months after baseline and the proportions of dietary and lifestyle factors achieved such as enjoyment of school life and dietary intakes assessed by FFQW82. Change from baseline (CFB) at 6 months was the primary endpoint. A linear mixed-effects model was applied. As for dietary intake, the treatment effect was estimated as an interaction term between baseline and treatment “baseline*treatment”.

**Results:**

The intention-to treat analysis included 951 (94.7%) and 1035 (89.8%) individuals in the SPRAT and control groups, respectively. The CFB in the 6-month SPS score adjusted for baseline was lower in the SPRAT group (-0.29) than in the control group (0.62), but the difference was not statistically significant -0.91 (*p* = 0.093).

**Conclusions:**

Although the primary endpoint tended to denote improvement in the SPRAT group compared to the control group, the improvement was not significant. Favourable effects were observed in some secondary outcomes and statistically significant treatment*baseline interactions were observed for several dietary intakes. These results imply that CFBs of dietary intake were increased or decreased in a favourable direction depending on the baseline intake, especially in the SPRAT group.

**Trial registration:**

UMIN000026715. (27/03/2017)

**Supplementary Information:**

The online version contains supplementary material available at 10.1186/s12889-022-12832-7.

## Background

Increases in mental health problems in adolescents are of major public concern [1–6], and developing effective and practical programmes to reduce subjective psychosomatic symptoms (SPS) is needed. Adolescents’ lifestyles have been associated with severe SPS [7, 8] and a systematic review showed the relationship between diet and mental health in adolescents [9]. Potential influence of diet on mental health among adolescents is significant. To support adolescents in attaining a healthy and satisfying life, health education, nutrition, and psychological support were highlighted as priorities [10].

Assessments of the effectiveness of interventions by targeting main lifestyle elements such as healthy dietary intake [11–13] and physical activities [14–20] were reported. Previously we developed a school-based home-collaborative dietary and lifestyle education programme (PADOK) that was proved to be more effective for the improvement of poor SPS scores than conventional classroom education by a cluster randomised controlled trial [21]. The PADOK attempted to change adolescents’ diet and lifestyles in a non-compulsory way, with dietary habits assessed by the Food Frequency Questionnaire with 82 food items (FFQW82) [22]. Because adolescents’ health behaviour is largely influenced by home-related factors, such as dietary intake and eating patterns at home, physical activity, and whether the family’s lifestyle is sedentary, parents/guardians are in a unique position to participate in dietary and lifestyle education programs. Parents/guardians have been shown to play an important part in their adolescents’ dietary and lifestyle education [23–27]. As an improvement to PADOK, we developed a school-based dietary and lifestyle education programme involving parents/guardians to reduce SPS in adolescents (SPRAT) [28]. The SPRAT programme focussed more on dietary education and more cooperation with parents/guardians than the previous effort because such involvement is necessary to implement effective dietary and lifestyle improvements in adolescents. This study evaluated the effectiveness of SPRAT to reduce poor SPS and improve healthy dietary intake in adolescents based on a cRCT.

## Methods

### Study design

This was an open-label, parallel group, school-based 6-month cRCT with individual middle schools as the unit of allocation and individual students as the unit of analysis. The design conformed with the Consolidated Standards of Reporting Trials (CONSORT) guidelines. Before recruitment, we registered the study with UMIN-CTR and uploaded the study protocol [28].

### Study setting

Voluntary middle schools in Kumamoto and Miyazaki Prefecture located in the south area in Japan.

### Participants

School was the unit for cluster randomisation. Participants were all eligible adolescents of these middle schools in school years 1 and 2 (age 12–14 years). Therefore, participants having both healthy and unhealthy attitudes and behaviour were mixed. The participants were registered between October 2018 and December 2019. Parents/guardians in the SPRAT group were asked to join the programme to help their children.

 JW and MW approached the Municipal Board of Education and the Principal Meetings in each area and got approval. Subsequently, JW identified volunteer middle schools and asked permission to enroll participants by telephone or a visit. Trial content was explained and efforts to obtain consent for schools’ cooperation were made. Several telephone meetings as well as face-to-face meetings with responsible persons and persons in charge of the Municipal Board of Education and the Meetings of Principals of Schools in each area were conducted. Further, we conducted several information sessions for parents/guardians in each school, for instance.

A minimum of 40 students was set as a requirement. Criteria for student participation are shown the study protocol and in Supplementary file 1. Written informed consent was obtained from all participants, including both students and their parents/guardians. As for participant recruitment and participant assignment, see the study protocol [28].

### Randomisation, allocation concealment and blinding

Randomisation was conducted using a permuted-block technique using a randomisation list. Schools had been allocated manually by random numbers obtained using a personal computer (PC). KY generated the allocation sequences and JW enrolled schools and assigned participants to interventions. Due to the nature of the treatment, participants could not be blinded to the type of dietary and lifestyle education. However, data management team members, except for the project coordinator and research assistants, were blinded to the group allocations. To minimise the risk of bias, we developed strict protocols for follow-up assessment procedures (in Japanese) and trained research assistants to adhere to these protocols.

### Interventions

The follow-up period was 6 months from randomisation (baseline). A 6-month intervention using SPRAT was conducted. The control was a usual school programme. This study was conducted according to the guidelines of the Declaration of Helsinki. The trial leader, a registered dietitian, provided training for the registered dietitians and learning support assistants based on the ‘registered dietitian training programme for SPRAT at the study management centres in Miyazaki and Kumamoto (around a 5-hour period). A summary of the intervention is as follows and details of the intervention are shown elsewhere [28].

#### SPRAT programme group (intervention group)

SPRAT is a dietary and lifestyle education programme of 6 monthly sessions involving parents/guardians to reduce SPS and to improve students’ health behaviour and dietary and lifestyle habits including physical activity and increase enjoyment of school life. Students are encouraged to increase intake of staple foods (rice, bread, etc.), main dishes (soy, fish, eggs and meat) and vegetables, particularly at breakfast and are assisted in developing skills in food selection by advice and education.

The FFQW82 questionnaire (at baseline and 6 months) [22], student’s SPS questionnaire (SPQ), and student’s Lifestyle Questionnaire (LQ) (at baseline and 2, 4, and 6 months) were administered. Participation self-check sheets (PPS) were provided to parents/guardians in the intervention group. Monthly sessions were conducted using a booklet ‘Smart Life’ which was delivered by registered dietitians and directed toward schoolteachers, students, and parents/guardians. Parent manuals (PPM) (every month) and a newsletter (4 times) were provided to parents/guardians in the SPRAT group. Thus, SPRAT also aimed to improve students’ *health* behavior and lifestyle habits as summarized in Table 4. Parents/guardians in the SPRAT group were required to participate regarding several issues according to the programme strategies. For instance, five homework assignments for students as well as parent manuals were distributed and practices to improve students’ lifestyle were conducted with their parents/guardians during the study period.

#### Usual programme (control group)

**Fig. 1:**
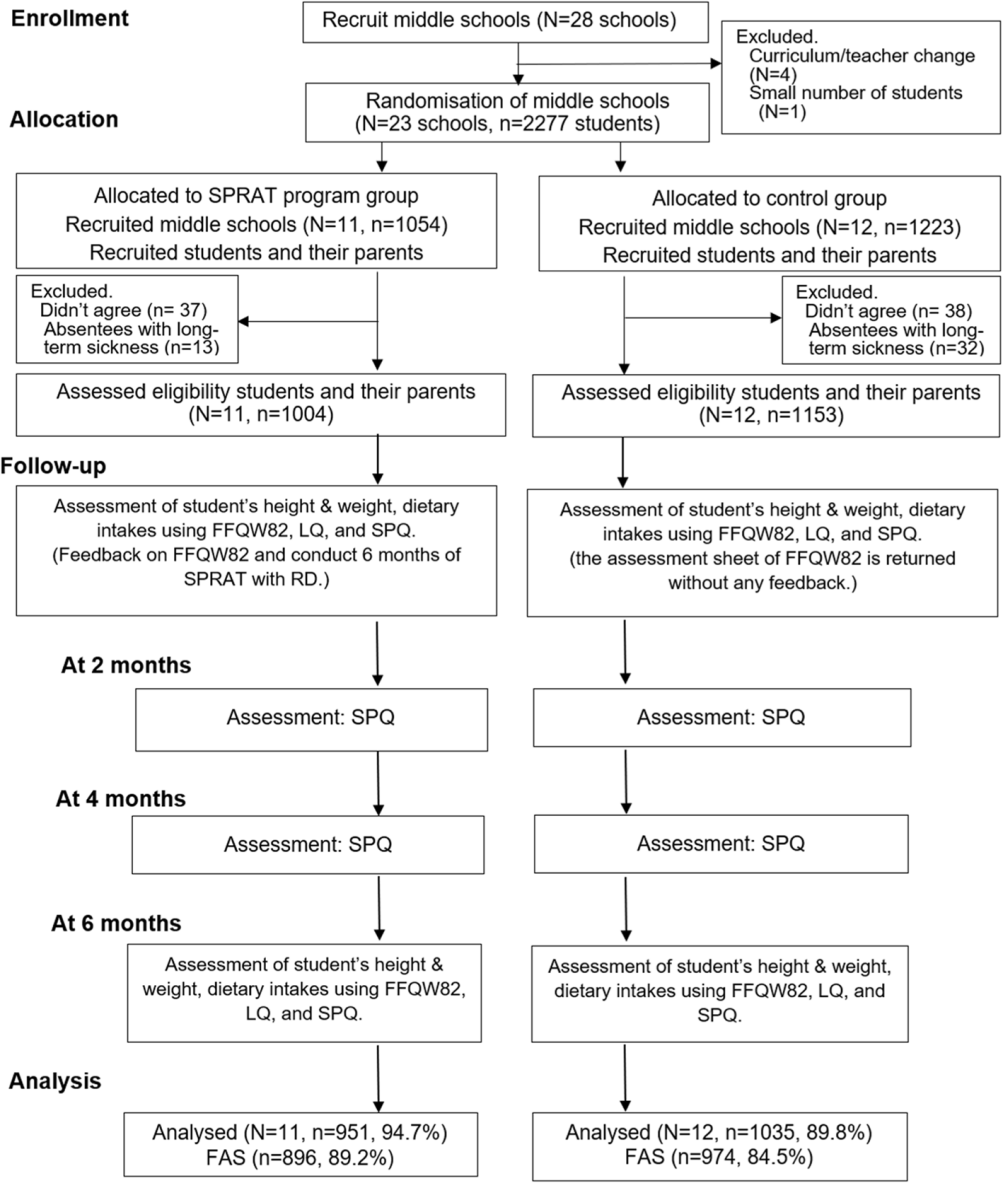
Flow diagram. CL, classroom lesson; FFQW82, Food Frequency Questionnaire with 82 food items; HW, homework; LQ, Lifestyle Questionnaire; Parents, parents/guardians; PM, parents’ manual; PPS, parent-participation self-check sheet for parents/guardians; RD, registered dietitian; SPRAT, School-based dietary and lifestyle education involving parents for reducing subjective psychosomatic symptoms in Japanese adolescents; SPQ, Subjective Psychosomatic Symptoms Questionnaire

Students in the control group participated in the schools’ usual programme in health education classes. That programme was composed of existing health curriculum about diet and/or exercise that was routinely taught at each participating school. The registered dietitian, learning support assistants, or the teacher assessed dietary intake using the FFQW82. Teachers in junior high schools distributed and collected questionnaires for parents/guardians as shown in Fig. 1.

### Study hypothesis

The hypothesis was that students who participated in the SPRAT group would have a greater decrease in their mean SPS score and more appropriate dietary intake than students in the control group after 6 months.

### Outcome measures and background variables

#### Primary outcome

The primary outcome was the SPS score at 6 months from baseline measured by responses to the SPQ, which consists of questions on nine symptoms such as fatigue, headache, lassitude, irritation, lack of concentration, lack of motivation, poor ability to wake up in the morning, upset stomach and stiff shoulder. Using a Likert scale (5 levels: ‘0 = never’ to ‘4 = always’), we calculated the SPS score (0–36 points) as the sum of the category values for the nine items. The validity and reliability of the SPS score were shown elsewhere [21]. The primary endpoint was the difference in the change from baseline at 6 months (±1 month) between the two groups.

#### Secondary outcomes

Secondary outcomes were the SPS score at 2 and 4 months from baseline, dietary and lifestyle factors (sleeping habits, eating habits, enjoyable school life, and physical activity), and dietary intake (11 energy intakes by food group and 9 nutrients) assessed by the FFQW82 [22] and body mass index (BMI) at 6 months from baseline. A school nurse measured BMI. The dietary intake standard [29] states that the target BMI range (≥18 years old) can be applied to investigate the intake and output in the energy balance in adults but not children. For junior high school students, a growth curve indicating the level of obesity based on the School Health Law is used. Therefore, in this program, SPRAT used this growth curve to perform an exercise to plot the current height and weight for the purpose of students recognizing their own physique and maintaining an appropriate physique.

On the other hand, the potential significance of this study is to reduce mental and physical health problems in the study participants 12 to 13 years old- in the transitional period from adolescence to adult, and to track this o risk to the next generation [30]. This also helps prevent health problems in the future.

For example, regarding the health risks to the next generation, according to the National Health and Nutrition Examination Survey [31], the percentages of obese people in their 20s was 26.8% for males and 5.7% for females and the percentages of the underweight individuals were 9.1% for males and 21.7% for females. In this study, we thought that it was meaningful to get BMI change as a reference value for changes in the body. Therefore, the BMI endpoint was used.

### Sample size

The required sample size was determined based on information needed to detect a difference in the primary outcome with a significance level of 5% and a power of 80%, under the assumptions of 40 students per cluster (same sample size for each cluster), an effect size (for severe SPS) of 0.3, and an intraclass correlation coefficient of 0.02. The effect size was estimated from our experience in a former study [21]. In total, participation by 28 schools (14 schools in each arm) was needed.

### Statistical analysis

We conducted the statistical analyses following the New Guidelines for Statistical Reporting as previously described [32].

We used descriptive statistics to assess the balance between the trial arms at baseline. Continuous variables were summarized as mean, standard error (SE), and intraclass correlation coefficient (ICC) estimated using nested analysis of variance (ANOVA) considering a cRCT study design. The difference between the two groups was examined based on an intention-to-treat (ITT) principle with the full analysis set (FAS). For the primary outcome, we defined the FAS as full data for baseline and endpoint (at 6 months) SPS scores. Change from baseline (CFB) at 6 months was the primary endpoint. Dietary and lifestyle factors were categorised as dichotomous variables. A general linear random-effects mixed model employing the restricted maximum likelihood method was used to analyse continuous variables. For dichotomous secondary outcomes, random effects logistic models (subject-specific models) were used, and associations were shown as odds ratios (ORs) and 95% confidence intervals (CI). Primary outcome measure was used to examine the effects of the intervention by a crude model (model 1), a model adjusted for baseline values (model 2), and a multivariate-adjusted model (adjusted for baseline, sex, age, and school type [private or public].) (model 3).

As for the secondary outcomes such as dietary intake, on the other hand, the treatment effect was estimated as a baseline*treatment interaction in those models that include a baseline*treatment interaction term. We expected the interaction term baseline*treatment to be statistically significant. Since the study participants were both underweight and overweight, to accept the validity of the interaction between the treatment and dietary intake it is reasonable to consider baseline intake. Participants were encouraged to consume either lesser or greater amounts in consideration of estimates based on baseline intake.

For missing values, the analyses by the imputation of missing data were performed using the last observation carried forward method (LOCF) and a multiple imputation method (MI) using chained equations under the assumption of missing at random [33].

All tests for significance were conducted using a two-sided approach with a 5% significance level. *P* values were shown only for the primary analyses and 95% CI was shown for the secondary analyses. All statistical analyses were performed using SAS V.9.4 for Windows (SAS Institute).

### Data management and monitoring

Personal information was coded and anonymised. All data and documents related to this study were managed as planned [28]. The ethics committee will receive a report at the conclusion of the study and after the final results.

### Protocol amendments

No amendments to the protocol or of the follow-up of adverse events.

### Ethics and dissemination

 The All tests for significance were conducted to the Declaration of Helsinki and the ethical guidelines for medical research on humans. This study was approved by the Ethical Review Board of the Medical Ethical Committee of Minami Kyushu University in 2017 (Number 137).

### Patient and public involvement

There was no patient or public involvement.

## Results

The study flow is summarised following CONSORT [34] in Fig. 1. Among the eligible schools, 5 were excluded from randomisation because of curriculum/teacher changes (4 schools) and too few students (1 school). In total, 23 middle schools (*n* = 2277 students) were recruited. Finally, 11 schools (*n* = 1004) in the SPRAT group and 12 schools (*n* = 1153) in the control group were randomised. Students who did not agree to enter the study (*n*= 75 students) or who were absent for long-term sickness (*n* = 45 students) were excluded. Participating in the analysis were 951 students in the SPRAT group (94.7%, 486 boys and 465 girls) and 1035 students in the control group (89.8%, 424 boys and 611 girls) (see Fig. 1; Table 1). One girls’ school was included in the control group. Two of 11 intervention schools and 3 of 12 control schools were private schools. Classroom lessons were conducted monthly as planned in the SPRAT group and intervention group. Details of the classroom lessons were described elsewhere [28]. The mean age, SPS scores, BMI, and dietary intake by meals, energy intake (kcal) by food groups and nutrients at baseline, proportions of sexes, school type, and dietary and lifestyle factors are summarised in Table 1. As for dietary and lifestyle factors, the proportion of the response of “very good” was summarised. At baseline, the mean values were similar among the SPRAT and control groups except for the SPS score and dietary and lifestyle factors of “Staple food consumed per breakfast”, “vegetables consumed per breakfast”, and “dairy products consumed per day” (Table 1).

**Table 1:**
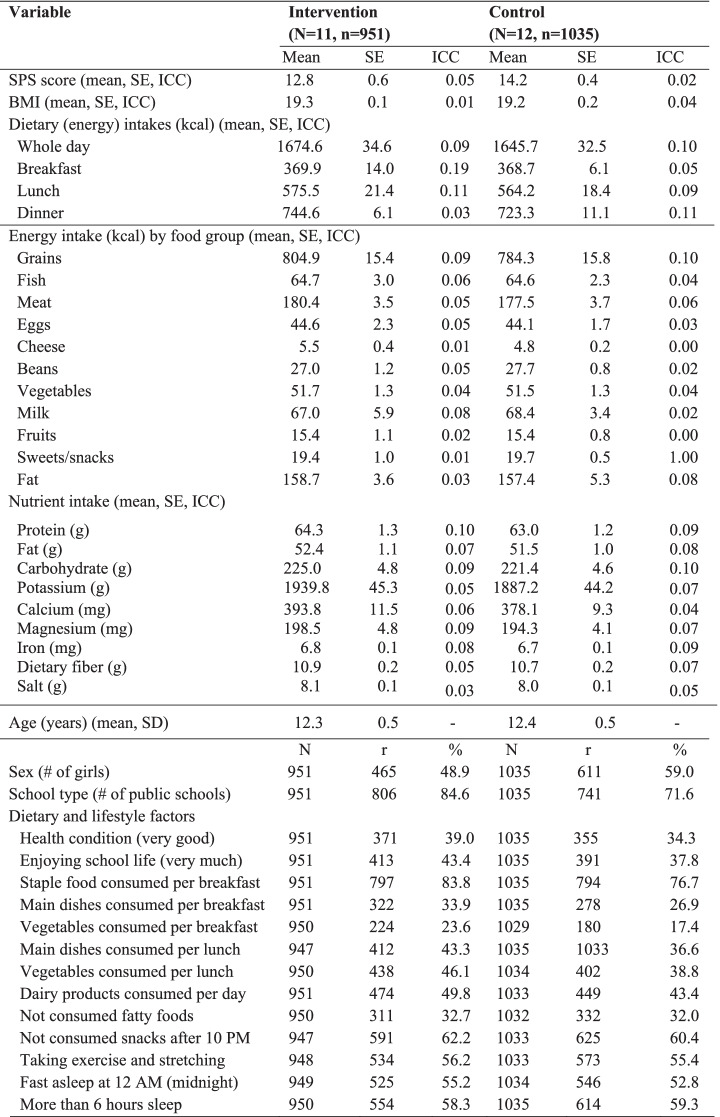
Summary statistics of the SPS score, BMI, and energy intake at breakfast, lunch, dinner, whole day, by food group, nutrient intake, and dietary and lifestyle factors at baseline. (*n* = 1986)

*SE *standard error, *SPS *subjective psychological symptoms, *BMI *body mass index, *ICC *intra-correlation coefficient, *r *number of responses, *Note:* SE was estimated by random-effects one-way ANOVA (cluster effects was treated as random-effects). Estimated using SAS NESTED procedure

Because interactions with time were significant, the analyses were conducted independently at various time points (2, 4, and 6 months). Using the mixed-effects model, the CFB in the 6-month SPS score was not statistically significant for crude (-0.40, 95%CI: -1.62 to 0.81, *p* = 0.497), baseline adjusted (-0.91, 95%CI: -1.99 to 0.17, *p* = 0.093), and multivariable adjusted (-0.89, 95%CI: -2.03 to 0.24, *p* = 0.116) values. Results of LODF and MI showed similar estimates (Table 2). Among secondary endpoints, lower SPS scores in the SPRAT group compared to the control group were observed in the CFB in the 4-month SPS score (baseline adjusted: -1.60, 95%CI: -2.87 to -0.33); multivariable adjusted: -1.64, 95%CI: -2.96 to -0.32) (Table 3). Dietary intakes with a non-significant baseline*interaction in the mixed-effects model were calculated using the mixed-effects model. Three of 11 energy intakes by food group (kcal) and 8 of 9 nutrient intakes had statistically significant interactions. Assessing baseline*treatment interaction becomes important for evaluating “the change in dietary behaviour” appropriately when considering the purpose of dietary education. (see Supplementary file 2) Further, using the random-effects logistic model, favourable effects on dietary and lifestyle factors were observed, such as “enjoying school life” (OR 2.01, 95%CI: 1.59 to 2.53), “staple food consumed per breakfast” (OR 1.49, 95%CI: 1.06 to 2.08), and “main dishes consumed per lunch” (OR 1.53, 95%CI: 1.08 to 2.16) (Table 4).

**Table 2:**
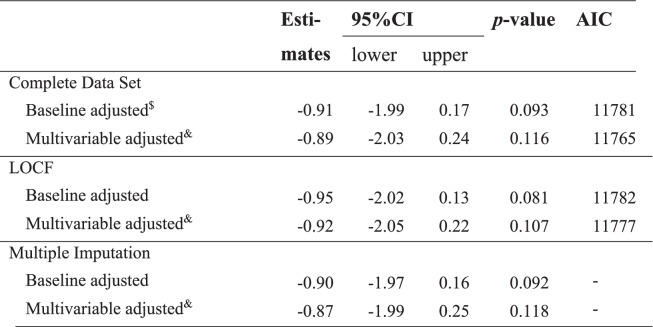
Mean SPS score at 6 months from baseline, results for primary endpoint and estimates of the mean difference between SPRAT and control groups by mixed-effects model. (*n* = 1872)

*CFB *change from baseline, *LOCF *last observation carried forward, *AIC *Akaike information criteria; $: interaction term for baseline and treat (*p* = 0.047), &: adjusted by baseline, sex age school founding entity [private or public]

**Table 3:**
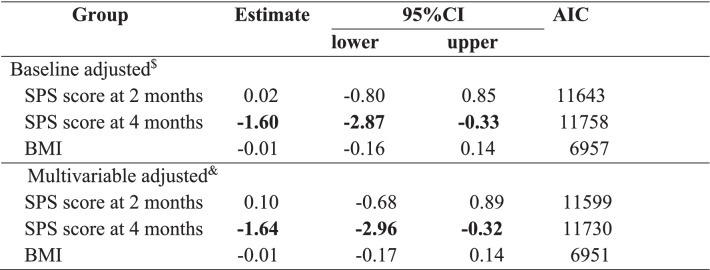
Estimates of the mean difference of the values of secondary outcomes (SPS score at 2 and 4 months, and BMI months from baseline) between SPRAT and control groups by mixed-effects model

*AIC *Akaike information criteria, *BMI *body mass index, $: adjusted for energy intake of whole day; &: adjusted for baseline, sex, age, school funding entity (private or public)

**Table 4:**
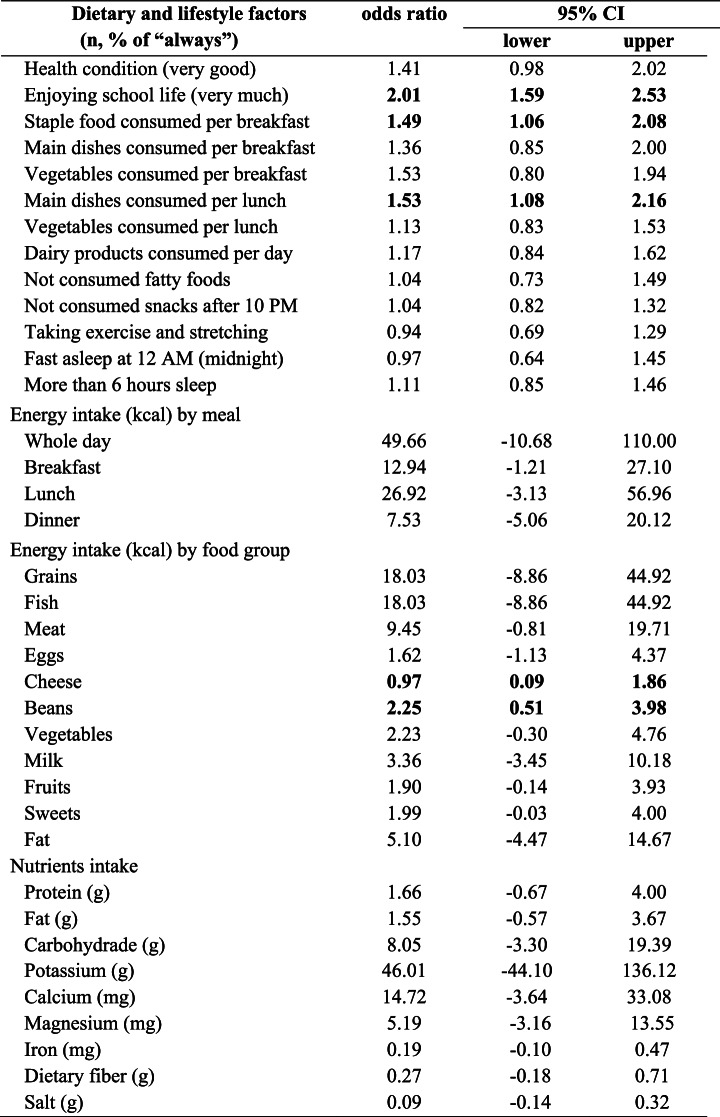
Effects of dietary and lifestyle intervention on the dietary and lifestyle factors analyzed by the mixed-effects logistic model and regression model (Multivariable odds ratio and multivariable adjusted regression coefficients, *n* = 1870)

Note: Multivariable adjusted: adjusted by baseline, sex, age, and school type. Estimates were based on Quad method (points=10) (SAS);   For the other estimates, those were almost similar among the estimated methods of residual PL, PL, and Quad (SAS)

.

## Discussion

### Statement of the principal findings

The SPRAT programme offered school-based dietary and lifestyle education involving parents/guardians of middle school students in Japan to reduce poor SPS and improve dietary intake. The trial tested our hypothesis that students who completed the SPRAT programme would have an improved SPS score, a more enjoyable school life, healthier dietary and lifestyle habits, including those related to diet and physical activity, and intake of healthier foods and nutrients. The CFB of the SPS score at 6 months adjusted for baseline was lower in the SPRAT group than in the control group but not with statistical significance. Only the CFB at 4 months differed significantly in the SPRAT group compared to the control group. Favourable effects were observed in some of the secondary outcomes. Especially, it was interesting that statistically significant baseline*treatment interactions were observed for many variables of dietary intake. These results imply that CFBs of dietary intake were increased or decreased in a favourable direction depending on the baseline intake, especially in the SPRAT group, where participants were encouraged to take appropriate nutrients from food based on baseline information.

Several explanations have been suggested for the mechanism of the connection between dietary and lifestyle interventions with psychosomatic symptoms [9, 35–37]. The present study offers clues on how to offer effective practical measures for minimising SPS and its potential influence among adolescents.

Compared to the PADOK, SPRAT reinforced the role of parents/guardians in changing dietary behaviour and reducing SPS in adolescents as described in the Method section. Although the effects of reducing SPS were weak except at 4 months, dietary behaviour and dietary intake, especially at lunch, were affected by parents/guardians to some degree. For instance, adolescents’ health behaviour was largely affected by factors at home such as dietary intake, eating patterns, physical activity, and a sedentary lifestyle [25]. Significantly better results of dietary and lifestyle interventions in overweight youth were achieved with parental involvement [26] Physically active parents were more likely to have physically active children in a systematic review [27]. Further, comprehensive behavioural family dietary and lifestyle interventions improved weight outcomes in youths in a meta-analysis [24]. Another review [23] noted the need for further studies on school-based interventions with parental components. The inclusion of participation by parents/guardians might have helped to obtain good effects as summarised in the report of a task force [38]. We conducted a subgroup analysis of the SPRAT group to obtain information about the SPS, BMI, and dietary intakes by concordance of the participation self-check sheet. (Results are not shown.) We calculated the average of those outcomes by the concordance level by classifying the concordance as GCR (concordance between parent/guardian and student for “very good”), NCR (concordance for “not very good”), and discordance. There were not large differences in the mean values of the outcomes at baseline, but at 6 months there seemed to be a tendency toward increases in the difference in dietary intakes for breakfast, lunch, dinner, and the whole day. These differences were twice as large for NCR compared to GCR, especially for responses to the questions “Taking exercise and stretching”, “Fast asleep at 12 AM (midnight)”, and “Not consumed snacks after 10 PM”. This tendency can be interpreted as the effects of the SPRAT both for parents/guardians and adolescents to some degree.

### Strengths and weaknesses of the study

The following can be considered as the strengths of the SPRAT programme. First, in the usual school life, disseminating health knowledge to parents/guardians was less frequent than with the SPRAT programme. Such regular stimulation through this education (see Table 1 in the protocol paper [28]) has impacted improvements through the SPRAT programme. Second, the self-check-off points (parents/guardians also did), which were particularly focused on important issues, may be practised even among parents/guardians with a tight timetable. Regular stimulation might have encouraged attention to important issues related to a child’s lifestyle and food intake. Third, the inclusion of short messages regarding scientific evidence of the merits of enriched breakfasts in textbooks and newsletters should have allowed parents/guardians to access such information. Fourth, multiple stimuli for parents/guardians could increase opportunities to touch the consciousness of parents/guardians. The design of the SPRAT programme provides such opportunities that are insufficient in current standard school education.

However, several limitations remained. Unlike the former studies [11–20], this study was conducted in Japanese middle schools. Therefore, generalisability is a limitation of the study design. The lack of blinding of the intervention to school teachers and registered dietitians was also a limitation.

A cluster randomised controlled trial to evaluate school-based education on changing adolescents’ dietary and lifestyle can be a powerful tool. However, in cluster randomisation with the school as a unit, we included all the students in spite of variations in health, differing from the usual clinical trials. Healthy adolescents brought about ceiling effects, and the results may tend to be conservative. In this study, the SPS score at 4 months denoted a significant improvement in the SPRAT group compared to the control group. We interpreted the results as showing that the effects of the SPRAT programme were limited to the short term or were affected by the ceiling effect to some degree. A recent article that examined the effects of school-based physical activity interventions on mental health in adolescents by a cRCT suggested a potential ceiling effect due to a smaller potential for improvement [39, 40]. In examining the overall study population, they found no effects. However, from a subgroup analysis that included those with the highest levels of psychological difficulties at baseline, favourable results were obtained [36]. Using our study data, we performed a subgroup analysis of participants with the highest SPS scores at baseline but did not obtain different results from those of the overall study population. We should interpret this finding carefully because there could be other factors such as regression to the mean. In addition, we used CFB. Analysis of covariance using change from baseline is the preferred general approach [41].

The educational objective of the SPRAT regarding diet was based on ​​encouraging appropriate amounts consumed but which allowed each student to determine based on information provided whether the amounts were within the desired range and were neither excessive nor insufficient. Indications personally given by the SPRAT programme were roughly eating staple foods, main dishes, side dishes, and dairy products for breakfast ); having staple foods, a main dish, side dish, and even milk at lunch; setting a specified time for snacks, and eating a midnight snack no later than 22:00 ; limiting sweets with 300 kcal or more to 3 times a week; drinking non-caloric drinks when thirsty; understanding that a desirable physical constitution is within ± 2SD of the average weight on the growth curve; not overusing oil dressing and mayonnaise, and having only one dish that uses oils and fats at a meal. The behavioural changes obtained from SPRAT were reflected in the red straight lines of total energy intake, breakfast and lunch energy intake, cereals, dairy products, proteins, fats, carbohydrates (see Supplementary file 2). It is highly possible that the less amount moved in the positive direction and the excess part moved in the negative direction with respect to the above desirable dietary content. In contrast, the blue line indicates that the individuals made changes regardless of the programme. The blue line reflects data for the control group. As in the intervention group, they increase intake of what they perceived to be less than the appropriate amount and decreased intake of what they perceived to be excessive. However, because the adolescents in the control group made changes without a programme that included parent/guardian input, the range of change was understandably smaller than that of the intervention group. The range of change in the intervention group was larger than that in the control group due to the educational effect (knowing the appropriate amount, knowing the target amount, and receiving support from others). Of interest was that the range of changes in lunch was more pronounced than for breakfast. Considering that students brought their lunch in one-third of schools, it was possible that the actions of parents/guardians resulted in increased or lost weight. Looking at the raw data, 50% (29.8 kcal) of the total energy of 59.2 kcal increased from the baseline was the energy consumed at lunch. The big change beyond the voluntary effects of the parents was brought as the educational effect for parents. The results that the odds ratio of the main dishes consumed per lunch was 1.53 (1.08 to 2.16) supported the above interpretation. However, we need further careful analyses and further study are required.

Excluding adolescents with mental health problems may have diluted the effect of the intervention. The reason why exclusion criteria included students with mental health issues was as follows. In usual school programmes, it is difficult to include adolescents with mental health issues because those students will not come to school and will not join the school curriculum. There were 3 adolescents who had mental health issues: 2 with orthostatic dysregulation and 1 with epilepsy and these students had long-term absences from school.

In this study, we focussed on energy intakes calculated from the FFQW82 as indicators of healthy eating. Though the FFQW82 has been validated, its use was a limitation because it is based on subjective reporting.

Through completion of the registered dietitian training programme for SPRAT, registered dietitians should successfully carry out the intervention through accurate and uniform dissemination of information. Only a few registered dietitians have taken dietary education courses at junior high schools as part of the current education system in Japan. In fact, no registered dietitian who participated in nutrition education at these study schools had taken dietary education courses. Our programme used tailor-made teaching materials for classroom sessions, homework, and communication with students, their parents/guardians, and registered dietitians. These materials helped with communications among them, and inclusion of parents/guardians had a favourable effect on improving adolescents’ dietary and lifestyle compared to the PADOK. The effectiveness of dietary and lifestyle education for adolescents by school teachers was shown [42] and cognitive-behavioural skills-building interventions by teachers can have a positively effect in several important areas for adolescents at risk for serious problems including SPS. A multifocal lifestyle intervention is very ambitious choice and change in nutrition might have been simpler with larger efficacy. We are also preparing for the digitisation of teaching methods and teaching materials for schools. Additionally, consideration of measures to foster participation by parents/guardians could make sense, as recommended [43]. Using a risk-group approach may be resulted in useful information and recommended at least in the follow-up of the trial group or in secondary analysis. For this issue, further study is warranted.

## Conclusions

Although the result for the primary endpoint tended to denote improvement in the SPRAT group compared to the control group, the improvement was not significant. Favourable effects were observed in some secondary outcomes. By the interaction term of dietary intake, our results imply that CFBs of dietary intake were increased or decreased in a favourable direction depending on the baseline intake, especially in the SPRAT group, wherein participants were encouraged to take the appropriate amounts of nutrients from food. Change in nutrition might bring simpler with larger efficacy among lifestyle education. Findings from this evaluation of SPRAT can address critical issues indeveloping practical and successful educational programmes to minimise SPS and increase the effectiveness of dietary education and potentially influence adolescents in ameliorating SPS. Further study is warranted on the methods of cluster randomisation for healthy adolescents and the effects of the inclusion of parent/guardian participation.

## Supplementary Information

**Additional file 1.**

**Additional file 2.**

## Data Availability

The datasets used and/or analysed during the current study are available from the corresponding author on reasonable request.

## Abbreviations

AIC: Akaike information criteria
ANOVA: Analysis of variance
CFB: Change from baseline
cRCT: Cluster randomised controlled trial
FAS: Full analysis set
FFQW82: Food frequency questionnaire with 82 food items
ICC: Intraclass correlation coefficient
ITT: Intention-to-treat
LOCF: Last observation carried forward method
LQ: Lifestyle questionnaire
MI: Multiple imputation
PADOK: Programme for adolescent of lifestyle education in Kumamoto
PM: Parent manuals
PPS: Parent-participation self-check sheets
SPS: Subjective psychosomatic symptoms
SPQ: SPS questionnaire
SPRAT: School-based dietary and lifestyle education programme involving parents/guardians to reduce SPS in adolescents
