# Supplementary material for: Effects of ‘SPRAT’ programme for dietary and lifestyle education to improve psychosomatic symptoms and dietary habits among adolescents: a cluster randomised controlled trial
Source: BMC Public Health. 2022 Mar 8;22:461. doi: 10.1186/s12889-022-12832-7 (PMC8903559; doi:10.1186/s12889-022-12832-7)
Supplement: Supplementary file 1 — Additional file 1. [file 12889_2022_12832_MOESM1_ESM.pdf]

Supplementary File 1. Participants and eligibility criteria

Participants and eligibility criteria

Inclusion criteria

Students in first and second grades (ages 12 to 14 years) and their parents/guardians (SPRAT group only) from Miyazaki Prefecture and Kumamoto Prefecture middle schools that had agreed to cooperate with the study.

Students and parents/guardians who freely consented to participate after demonstrating sufficient understanding of the study after receiving a detailed explanation of its aims and procedures.

Students and parents/guardians who agreed to cooperate with the trial and could comply with the procedures related to the trial according to the instructions of the researcher or the teacher.

Exclusion criteria

Students who had mental health disorders; students who had repeatedly refused to attend school in the past; students who were judged unsuitable for participation in the trial due to health reasons cited by the school nurse; students who were judged inappropriate to participate in the trial by teachers and parents/guardians for the reason such as refusal to attend school.
