# Supplementary figures and images for: Effects of ‘SPRAT’ programme for dietary and lifestyle education to improve psychosomatic symptoms and dietary habits among adolescents: a cluster randomised controlled trial
Source: BMC Public Health. 2022 Mar 8;22:461. doi: 10.1186/s12889-022-12832-7 (PMC8903559; doi:10.1186/s12889-022-12832-7)

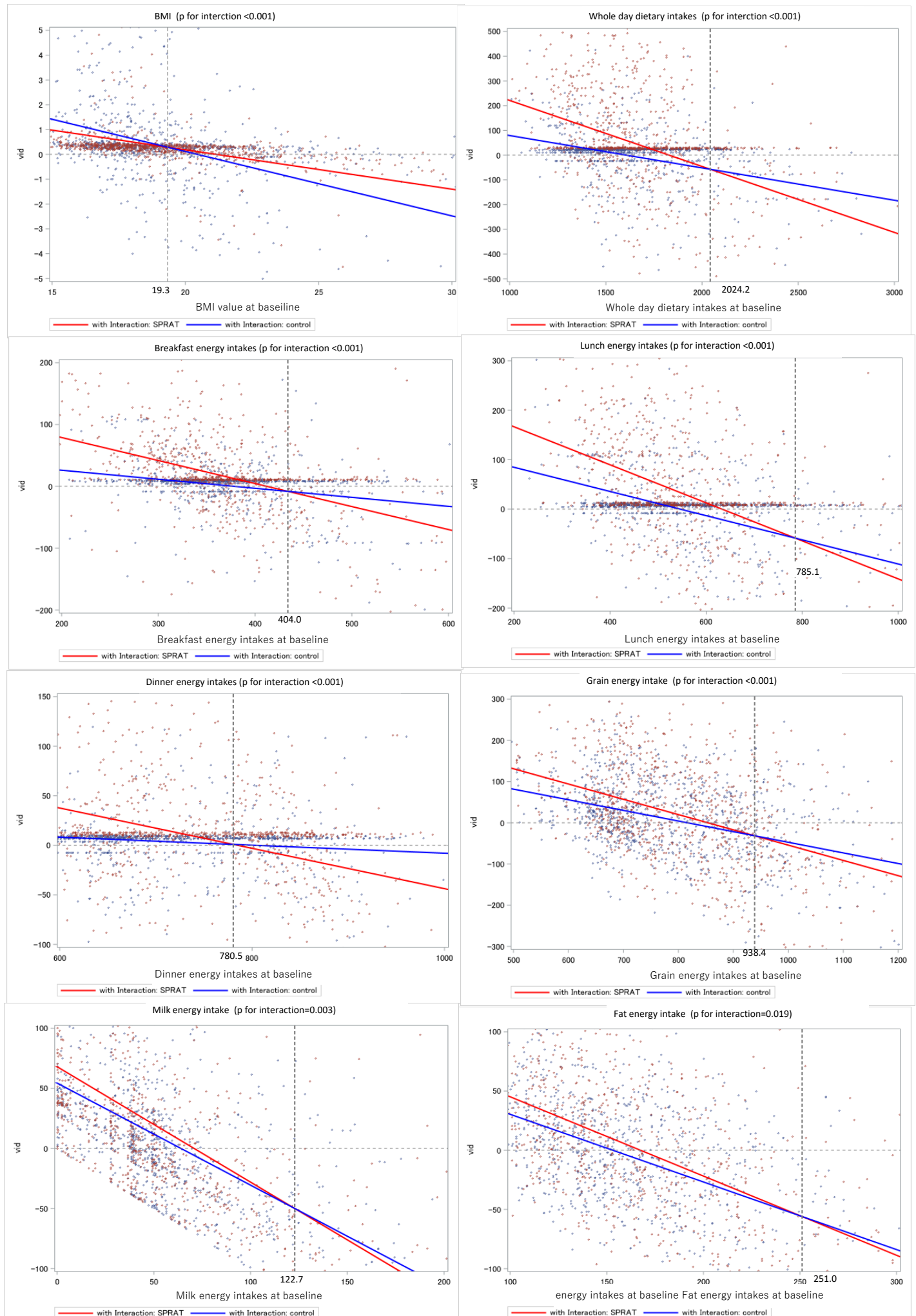

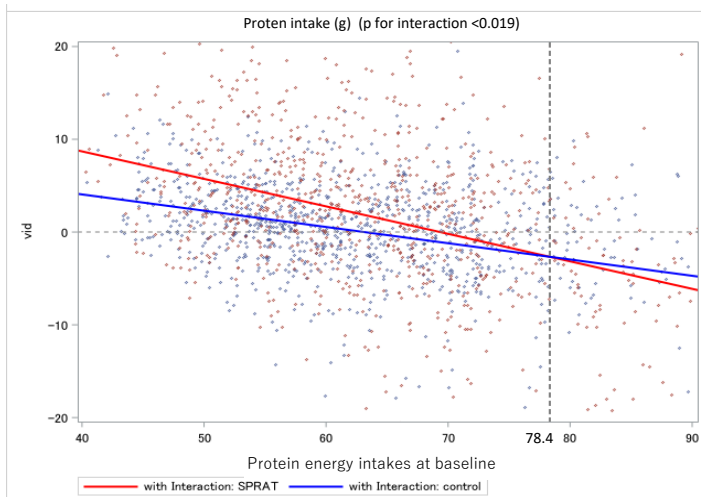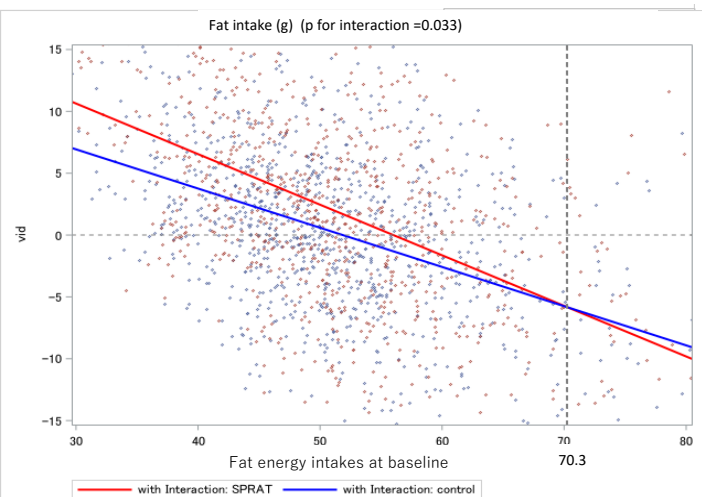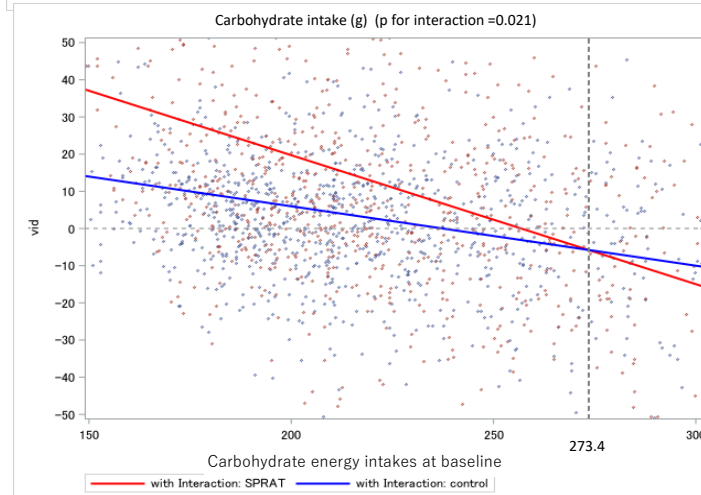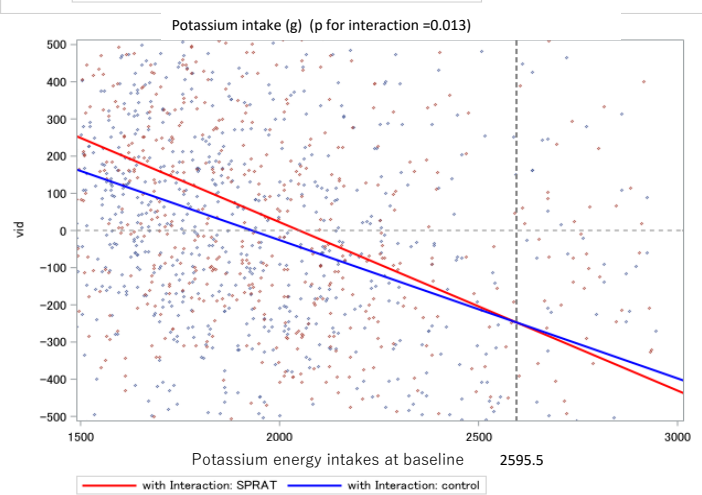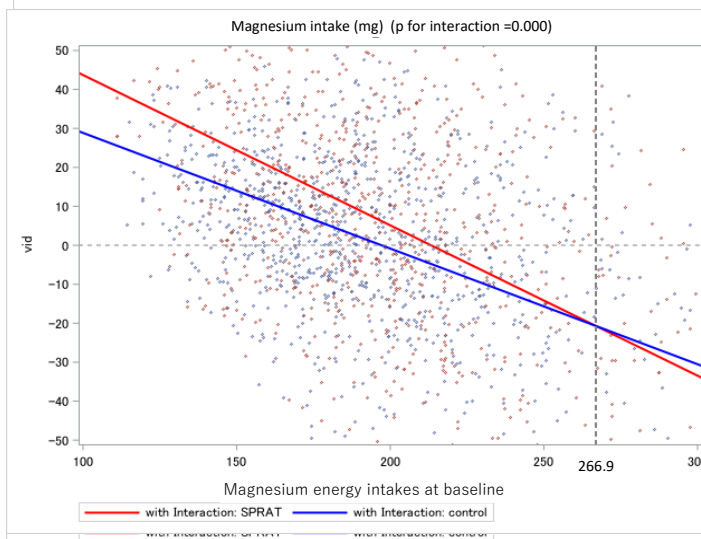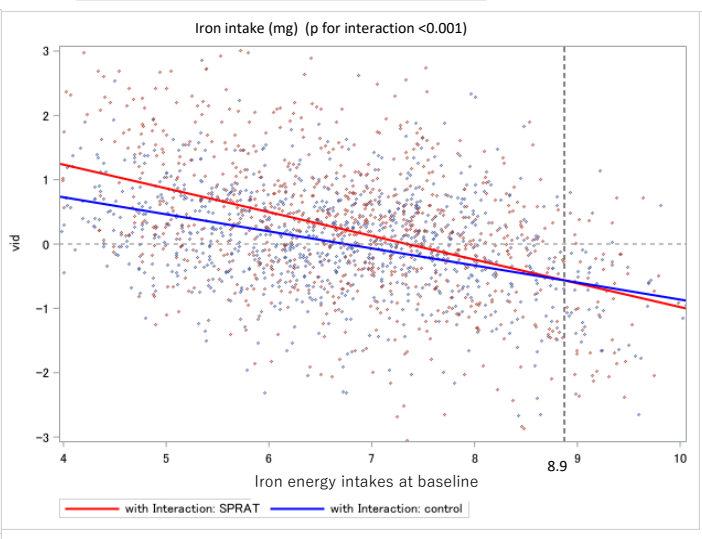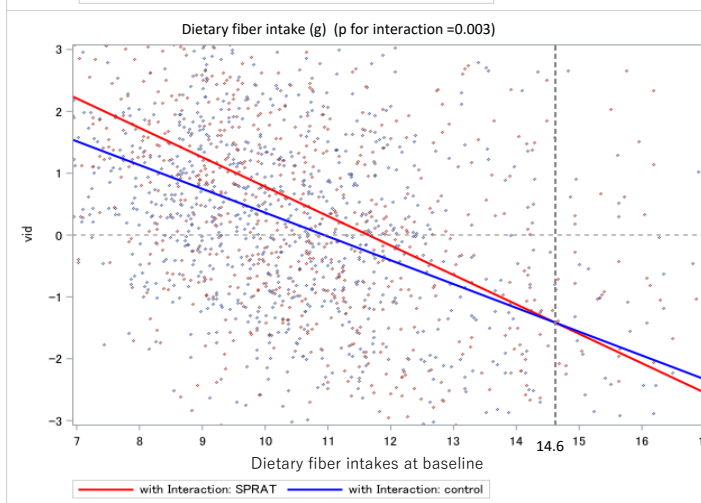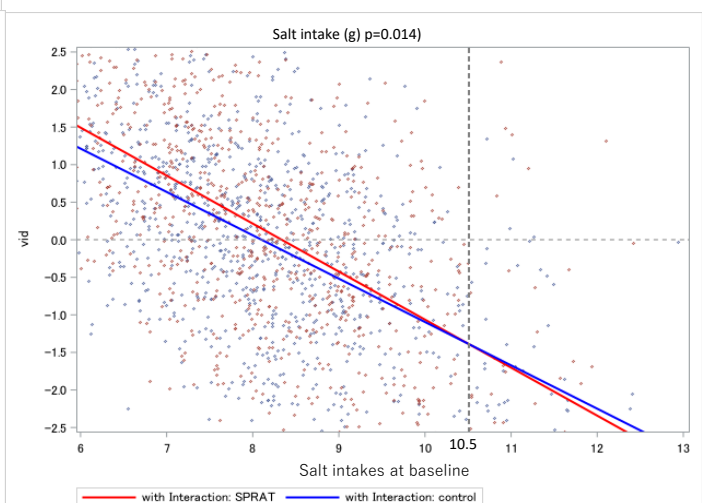

Supplement: Supplementary file 2 — Additional file 2. [file 12889_2022_12832_MOESM2_ESM.pdf]
